# NMU Is a Poor Prognostic Biomarker in Patients with Lung Adenocarcinoma

**DOI:** 10.1155/2021/5031479

**Published:** 2021-07-12

**Authors:** Yan Tang, Chunsheng Hu

**Affiliations:** College of Pharmacy (International Academy of Targeted Therapeutics and Innovation), National & Local Joint Engineering Research Center of Targeted and Innovative Therapeutics, Chongqing Key Laboratory of Kinase Modulators as Innovative Medicine, Chongqing University of Arts and Sciences, Chongqing 402160, China

## Abstract

Lung adenocarcinoma (LUAD) is the most prevalent histologic type of lung cancer, associated with a high incidence rate and substantial mortality rate worldwide. Accumulating evidence shows that the aberrant expression of neuromedin U (NMU) contributes to the initiation and progression of cancer. Herein, we explored whether NMU could be adopted as a new diagnostic and therapeutic marker in LUAD. The UALCAN and GEPIA web resources were employed to assess data on the NMU expression in LUAD. The STRING web resource was used to develop the PPI (protein-protein interaction) network of NMU, whereas Cytoscape was applied for module analysis. The Gene Ontology (GO) and the Kyoto Encyclopedia of Genes and Genomes (KEGG) analyses of NMU and the interacting proteins were examined using the WebGestalt tool. Survival analysis was performed with the Kaplan-Meier plotter tool. Results revealed that the NMU expression in LUAD was significantly higher than in the nonmalignant tissues. Moreover, higher NMU levels were dramatically related to shorter overall survival, first progression survival, and postprogression survival. The specific gene mutations G45V, R143T, and F152L of NMU occurred in LUAD samples and were associated with a worse prognosis in patients. KEGG and western blot analyses demonstrated an association of NMU with the cell cycle and the cAMP signaling cascade. Bioinformatic analysis and the in vitro experiments implicated NMU as a promising prognostic signature and treatment target for LUAD.

## 1. Introduction

Lung cancer is among the malignant tumors associated with high incidence and mortality rates worldwide [1]. Lung adenocarcinoma (LUAD) is the most prevalent histologic type of lung cancer that accounts for about 40% of lung cancer cases [2]. Over the past decade, great advancements in the treatment of patients with lung adenocarcinoma have been made, including surgery, radiotherapy, chemotherapy, or targeted therapy [3]. However, the prognosis of relapsed patients with LUAD remains poor. Recent studies have revealed the potential of targeted therapy in the repression of the growth of lung cancer cells by inhibiting the activation of key oncogenic molecules which drive LUAD progression [4–6]. Although targeted therapy yields promising results in early treatment, the development of drug resistance is linked to treatment failure [7]. There is a need to uncover critical genes and signaling cascades that mediate tumor progression of LUAD to develop advanced therapeutic strategies for LUAD management.

Several novel prognostic and therapeutic biomarkers for LUAD have been recently identified through bioinformatic analysis. For instance, Sun et al. reported higher expression levels of transcription factors (E2F1, E2F2, E2F5, E2F6, E2F7, and E2F8) in LUAD tissues than in normal lung tissues. Furthermore, bioinformatic analysis revealed that high transcription levels of these transcription factors were associated with poor prognosis in LUAD patients [8]. Elsewhere, Lu et al., through integrated bioinformatic analysis, revealed a reduction in the expression of tripartite motif-containing 56 (TRIM56) in LUAD and were associated with dismal prognosis. In addition, the increased TRIM56 expression repressed the migration and infiltration of LUAD cells [9]. A study by Tang et al. reported a remarkable overexpression of maternal embryonic leucine zipper kinase (MELK) in lung cancer which was inversely linked to the survival of LUAD patients following the bioinformatic analysis [10]. Additionally, Wang et al. identified five key genes (CCNB1, MAD2L1, CDC20, BUB1B, and TTK) linked to worse prognosis based on the bioinformatic analysis [11]. Therefore, bioinformatic analysis of various datasets can assist in the determination of several valuable genes in the prediction of a new prognostic signature and prospective treatment target. However, it is unclear whether these predictions via bioinformatic analysis accurately reflect the real situation in vivo. There is a need for verification of the results of bioinformatic analysis via biochemical experiments.

Neuromedin U (NMU) neuropeptide is expressed in numerous organs and tissues and plays diverse physiological and pathophysiological roles including contraction of smooth muscles, the balance of energy, and tumorigenesis [12]. Recent studies have implicated NMU as a key gene in the prediction of disease status and prognosis in cancers [13–15]. However, the role of NMU in LUAD remains elusive. Much more details on NMU, such a critical gene, in clinical application value with LUAD should be explored. Herein, we purposely explore the expression level of NMU in LUAD and its potential clinical application value.

## 2. Materials and Methods

### 2.1. Analysis of the NMU Expression in LUAD

The data from the TCGA data resource was used to explore the NMU expression via the UALCAN web resource (http://ualcan.path.uab.edu) [16]. Based on the UALCAN web resource, the NMU transcription level was evaluated in different cancer subgroups including cancer stages, race, smoking habit, and TP53 mutation status. Furthermore, GEPIA web resource (http://gepia.cancer-pku.cn/) [17], an interactive web tool, was employed to validate the NMU expression level between LUAD and nonmalignant tissue from TCGA and GTEx with a *p* value ≤0.05 and ∣log2FC | ≥2.

### 2.2. Survival Analysis of NMU in LUAD

We enrolled 719 LUAD patients for the survival analysis. The Kaplan-Meier plot (https://kmplot.com) [18], an online data resource, was applied to assess the OS (overall survival), FPS (first progression survival), and PPS (postprogression survival) differences between high and low NMU expression groups stratified according to the median expression score. *p* < 0.05 denoted statistical significance.

### 2.3. Pathway and Gene Ontology Enrichment Analysis

We used the STRING web resource (http://www.stringdb.org) [19] in the prediction of the PPI (Protein-protein Interaction) network whereby an interaction score > 0.9 denoted a significant interactive relationship. The PPI network was evaluated via the Cytoscape software package (version 3.7.2). Furthermore, WebGestalt (WEB-based GEne SeT AnaLysis Toolkit) online toolkit [20] was employed to enrich the KEGG pathway and gene ontology of all the genes that interacted with NMU in STRING.

### 2.4. Mutation Analysis

The CBioPortal web resource (https://www.cbioportal. org/) [21] with the data on DNA mutations, methylations, and gene amplification was employed to establish the association of genetic mutations with the initiation of LUAD. Using the cBioPortal web resource, we explored the top 10 genes that interacted with NMU. In addition, the OS and PFS (progression-free survival) were examined between NMU with alternation and without alternation.

### 2.5. Cell Lines and Antibodies

Lung adenocarcinoma cells HCC827, H1975, H1650, and A549 and nonmalignant lung cells 16HBE were purchased from Nanjing Cobioer Co., Ltd. HCC827, H1975, and H1650 were cultured in RPMI-1640 medium enriched with 10% FBS and allowed to grow under 37°C and 5% CO_2_ conditions. However, A549 and 16HBE were cultured in F12K and DMEM/high glucose medium enriched with 10% FBS under 37°C and 5% CO_2_ conditions. We purchased the antibodies of NMU (Affinity, Cat No. DF4238), p-Erk1/2 (Cell signaling technology (CST), Cat No. 4370), Erk1/2 (GeneTex, Cat No. GTX59618), p-FoxO3 (CST, Cat No. 13129), FoxO3 (CST, Cat No. 2493), cyclin A2 (CST, Cat No. 4656), cyclin B1 (CST, Cat No. 12231), cyclin D1 (CST, Cat No. 2922), P21 (CST, Cat No. 2947), P27 (CST, Cat No. 3686), and beta-actin (CST, Cat No. 58169).

### 2.6. RNA Extraction and qRT-PCR

The TRIzol Reagent (Cat No.15596026, Invitrogen) was employed to extract total RNA as described by the manufacturer. Then, cDNAs generated from 1 *μ*g of the total RNA were subjected to qRT-PCR using TB Green™ Premix Ex Taq™ II (Cat No. RR820B, Takara). The following primers were used for real-time PCR: NMU_F (5′AGCTCGTTCCTCACCTGCATGA3′), NMU_R (5′CTGCTGACCTTCTTCCATTCCG3′), beta-actin_F (5'CACCATTGGCAATGAGCGGTTC3′), and beta-actin_R (5′AGGTCTTTGCGGATGTCCACGT3′). Fluorescence emitted by SYBR was examined via the LightCycler96 detection system (Roche Molecular Systems, Inc.). The relative mRNA level of NMU was quantified and standardized to beta-actin mRNA expression and denoted as the fold change.

### 2.7. Western Blot

The RIPA lysis buffer (Cat No. P0013B, Beyotime Institute of Biotechnology, Jiangsu, China) was enriched with a protease cocktail inhibitor (Cat No. 4693132001, Roche) that was employed to isolate total proteins of the cells. The quantities of the isolated proteins were determined using the BCA protein assay kit (Cat No. P0009, Beyotime Institute of Biotechnology, Jiangsu, China). Thereafter, 20 *μ*g of total proteins was fractionated on a 12% SDS-PAGE gel and transfer embedded onto 0.22 *μ*M PVDF membranes (Cat No. ISEQ00010, EMD Millipore). Subsequently, the membranes were blocked with 5% nonfat milk at room temperature (RT) for two hours and then incubated overnight at 4°C overnight with the following specific primary antibodies: anti-NMU (1 : 200), anti-p-Erk1/2(1 : 1000), anti-Erk1/2(1 : 1000), anti-p-FoxO3 (1 : 1000), anti-FoxO3 (1 : 1000), anti-cyclin A2 (1 : 1000), anti-cyclin B1 (1 : 1000), anti-cyclin D1 (1 : 1000), anti-P21 (1 : 1000), anti-P27 (1 : 1000), and anti-beta-actin (1 : 5,000).After that, the membranes were incubated with the corresponding IRDye® 800CW Goat anti-Rabbit IgG (H + L) (1 : 15,000; Cat No. 926-32211; LI-COR) or IRDye® 680RD Goat anti-Mouse IgG (H + L) (1 : 15,000; Cat No. 926-68070; LI-COR) at room temperature (RT) for a further one hour. An Odyssey® CLx imaging system (LI-COR) was employed to capture blot images.

### 2.8. shRNA and Transfection

The shRNA was used to inhibit the NMU expression. We used the following sequences: NMU (5′CCGGCACAGAGCAATGCTATGGAATCTCGAGATTCCATAGCATTGCTCTGTGTTTTTTG3′). The control shRNA (Luciferase shRNA, shLuc) was obtained from Sigma-Aldrich (Merck KGaA). The 293T cells were used to package lentiviruses containing shRNA vectors. A549 cells were inoculated into six-well culture plates at 5 × 10^5^ cells/well. The culture medium was replaced by lentiviral particles mixed with polybrene (Cat No. TR-1003-G, Sigma-Aldrich; Merck KGaA) for 24 hours. Subsequently, the cells were selected for stable integration with 4 *μ*g puromycin dihydrochloride for 2 weeks (2 *μ*g/ml; Sigma-Aldrich; Merck KGaA).

### 2.9. BrdU Assay

Cell proliferation was explored using the BrdU incorporation assay. Glass coverslips in 24-well plates were inoculated with A549, A549-shLuc, or A549-shNMU cells and incubated for 24 hours. After that, the growth medium was replaced with a BrdU incorporation medium after 6 hours. The coverslips were incubated with mouse anti-BrdU antibody (Cat No.5292S, Cell signaling technology) overnight at 4°C. The coverslips were rinsed and incubated with Alex Flour 594-labeled anti-mouse IgG (Cat No. A-21203, ThermoFisher) for 1 hour at room temperature. Nuclei were labeled with DAPI. Images were captured using an Olympus fluorescence microscope (model: IX73, Tokyo, Japan). The numbers of BrdU-positive cells was counted from 5 random fields.

### 2.10. Colony Formation

The A549, A549-shLuc, or A549-shNMU cells were cultured in a 6-well plate (1,000 cells/well) for 2 weeks. The cells were subsequently stained with 0.5% crystal violet for 2 h at room temperature. The ImageJ software (V.1.52a; NIH) was employed to quantify the clones.

### 2.11. Statistical Analyses

All statistical analyses were conducted using GraphPad Prism V.7.0 (GraphPad Software, Inc.). Data were represented as the mean ± standard deviation, and the differences between group were analyzed using Student's *t*-test. *p* < 0.05 denoted statistical significance.

## 3. Results

### 3.1. The Expression of NMU in LUAD Patients

**Figure 1:**
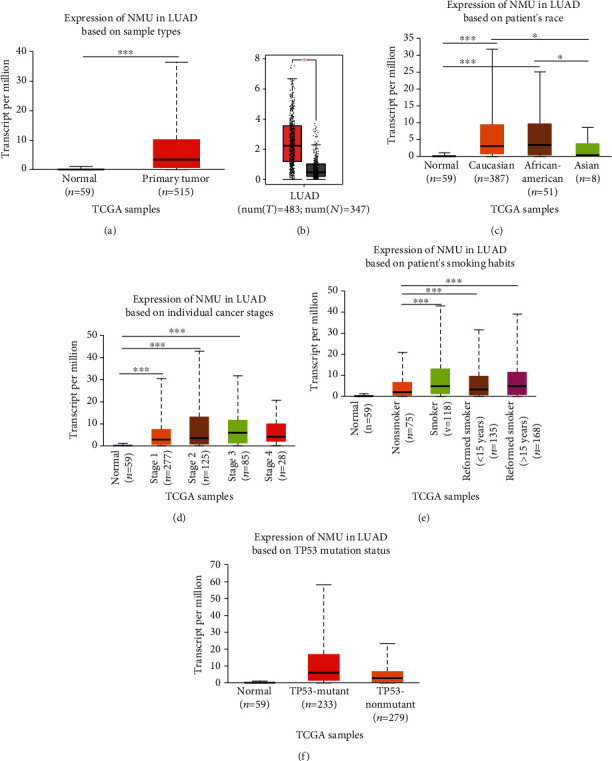
The expression of NMU in patients with LUAD. (a) The expression of NMU in LUAD as evaluated in the UALCAN database (^∗∗∗^*p* < 0.001 vs. normal). (b) The expression of NMU in LUAD, evaluated using the GEPIA online tool (^∗^*p* < 0.05 vs. normal, T: tumor; N: normal). (c) Expression of NMU in LUAD based on race (^∗^*p* < 0.05 vs. Asian group, ^∗∗∗^*p* < 0.001 vs. normal), (d) individual cancer grade (^∗∗∗^*p* < 0.001 vs. normal), (e) patient's smoking habits (^∗∗∗^*p* < 0.001 vs. nonsmoker), and (f) TP53 mutation status (^∗∗∗^*p* < 0.001 vs. nonmutation).

The UALCAN and GEPIA web resources were employed to explore the relative NMU expression between lung adenocarcinoma tissues and nonmalignant lung tissues. The results showed that NMU was remarkably elevated in LUAD tissues than in nonmalignant tissues (*p* < 0.001, Figures 1(a) and 1(b)). Furthermore, the association of the NMU expression levels with clinicopathological features of LUAD patients was explored via the UALCAN data resource. The results revealed elevated NMU expression levels in Caucasian and African-American patients relative to Asian patients (*p* < 0.05) (Figure 1(c)). The NMU expression gradually increased from grade 1 to grade 3 but noticeably decreased in grade 4. These results demonstrated that NMU was partly associated with LUAD grades (Figure 1(d)).  Figure 1(e) illustrates a remarkable increase in the NMU expression in patients with smoking or reformed smoking in contrast with that in patients with nonsmoking. In addition, LUAD patients with TP53 mutation expressed higher levels of NMU than those without TP53 mutation (Figure 1(f)).

### 3.2. High NMU Expression Level Is Associated with Unfavorable Survival in LUAD Patients

**Figure 2:**
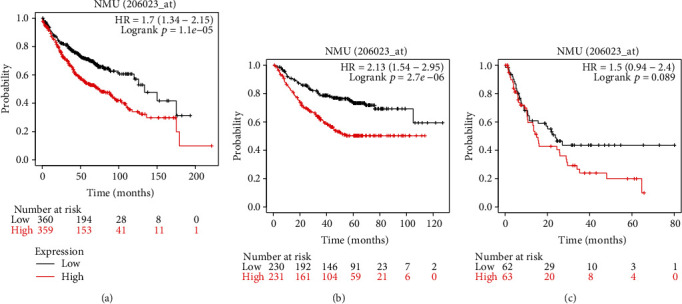
Higher expression of NMU is associated with poorer OS (a), FPS(b), and PPS(c) in LUAD patients.

In survival analysis, the Kaplan-Meier plot was used to determine the relationship of the NMU expression level with the fate of LUAD patients. Based on the OS curves, LUAD patients who exhibited high NMU expression were characterized by a remarkably low survival probability (HR = 1.7, 95%CI = 15.4 − 2.95, *p* = 0.0074) (Figure 2(a)). Furthermore, the FPS and PPS curves revealed that the elevated NMU expression reflected a dismal prognosis. Elevated NMU contents were associated with dismal FPS (HR = 2.13, 95%CI = 1.54 − 2.95, *p* = 0.0067) and PPS (HR = 1.5, 95%CI = 0.9 − 2.4, *p* = 0.089), respectively (Figures 2(b) and 2(c)). Overall, survival curve analysis demonstrated that the high NMU expression was suitable in the prediction of worse prognosis in LUAD patients.

### 3.3. PPI Network and Process Enrichment Analysis of NMU

**Figure 3:**
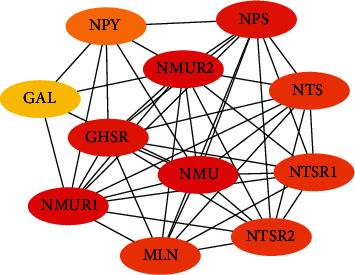
PPI network of the top 10 interacted genes related to NMU.

**Figure 4:**
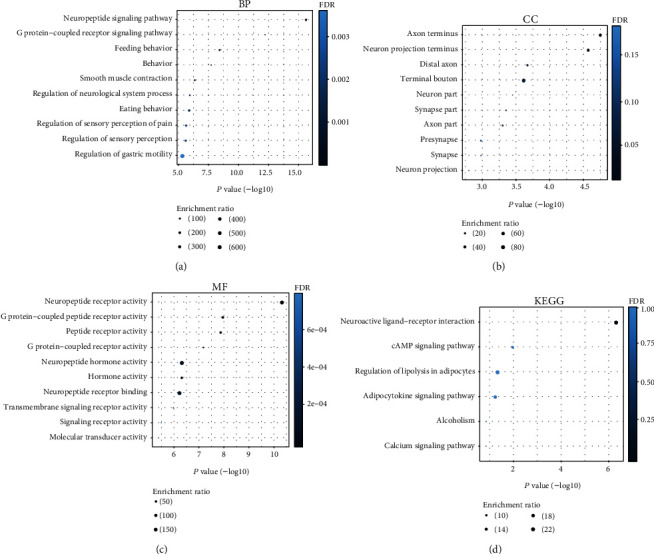
GO and KEGG pathway analysis of NMU and its interacted genes in WebGestalt. Top 10 GO and KEGG enrichment analyses of DEGs were displayed. (a) Biological process (BP). (b) Cellular component (CC). (c) Molecular function (MF). (d) KEGG pathway. FDR < 0.05 was considered significant.

The PPI network demonstrated the crosstalk between NMU crosstalk and the following functional genes: GAL (galanin peptides), NPY (proneuropeptide Y), NMUR1 (neuromedin U receptor 1), NMUR2 (neuromedin U receptor 2), GHSR (growth hormone secretagogue receptor type 1), NPS (neuropeptide S), MLN (promotilin), NTS (neurotensin/neuromedin N), NTSR1 (neurotensin receptor type 1), and NTSR2 (neurotensin receptor type 2) (Figure 3). The prospective function of NMU in LUAD, GO, and KEGG enrichment analysis was explored using WebGestalt. The BP (biological process), CC (cellular component), and MF (molecular function) of the genes involved in the top 10 were analyzed (Figures 4(a)–4(c)). The data demonstrated that those genes were linked to biological process, including neuropeptide signaling cascade, G protein-coupled peptide receptor signaling cascade, and feeding behavior. KEGG enrichment results revealed that the genes in modules 1-2 were primarily abundant in neuroactive ligand-receptor crosstalk and cAMP signaling cascade (Figure 4(d)).

### 3.4. Specific Mutations of NMU Genes in LUAD Patients

**Figure 5:**
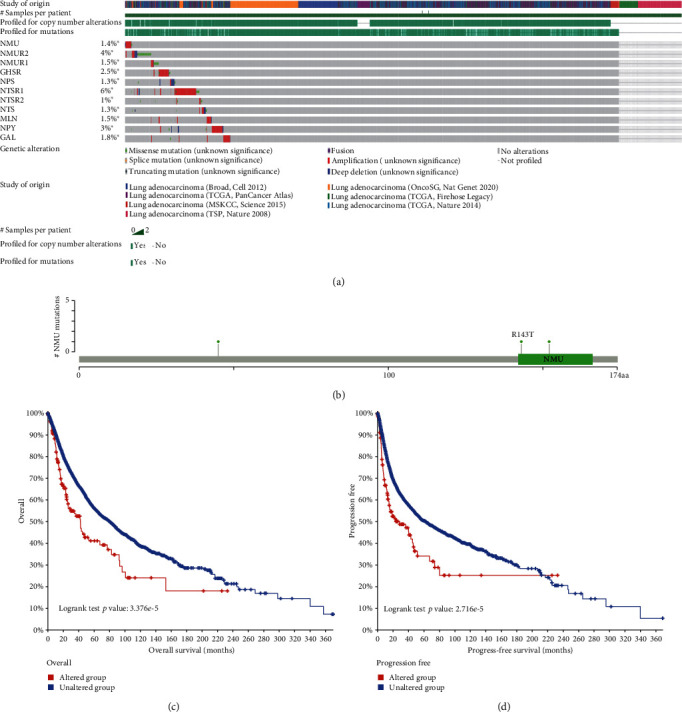
CBioPortal database analysis of the mutation of NMU and its interacted genes. (a) A visual summary of genetic alterations shows the genetic alteration of NMU and its interacted genes. (b) The particular mutations (G45V, R143T, and F152L) of NMU in LUAD patients. (c) Overall survival Kaplan-Meier estimate. (d) Progression-free survival Kaplan-Meier estimate.

The mutations of NMU and interactive genes (GAL, NPY, NMUR1, NMUR2, GHSR, NPS, MLN, NTS, NTSR1, NTSR2) were analyzed by the cBioPortal database. The results showed that the NMU gene-altered was 1.4% in 2068 patients with LUAD, and its interacted gene alterations were shown in Figure 5(a). Of note, there were 3 specific mutations G45V, R143T, and F152L of NMU in the LUAD samples (Figure 5(b)). Furthermore, the OS and PFS (progress-free survival) curves showed a dramatically higher survival probability in LUAD patients without NMU alterations than those with the NMU alterations (Figures 5(c) and 5(d), *p* < 0.05). Altogether, these results suggested that NMU gene mutation could influence the prognosis of LUAD patients.

### 3.5. In Vitro Validation of the NMU Expression

**Figure 6:**
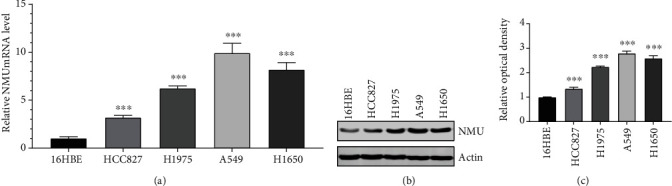
The high NMU expression in LUAD cell lines. (a) qRT-PCR detection of the levels of NMU in LUAD cells and lung normal cells. (b) Western blot analysis to detect the expression of NMU protein in LUAD cells and lung normal cells. (c) The bar chart showing the ratio of the NMU protein to actin by densitometry in the different cells. (^∗∗∗^*p* < 0.001 vs. normal 16HBE cell).

The NMU expression was validated at the mRNA and protein level in lung adenocarcinoma cells and nonmalignant lung cell line via qRT-PCR and western blotting. The data illustrated that the mRNA and protein contents of NMU in lung adenocarcinoma cells were higher in contrast with that of nonmalignant lung cells (Figure 6).

### 3.6. Effect of NMU on Cell Proliferation and Growth

**Figure 7:**
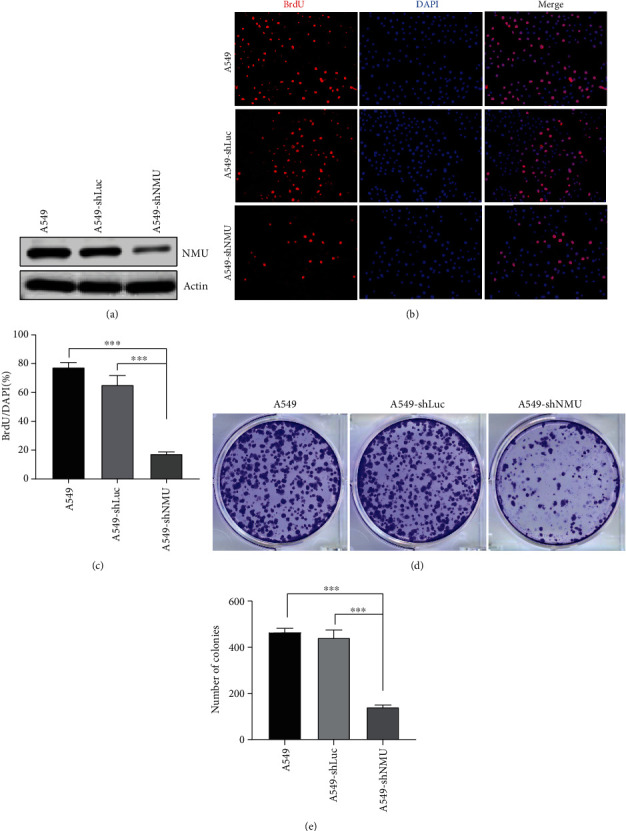
Knockdown of NMU inhibits LUAD cell proliferation and clone formation. (a) A549 cells are transfected with indicated shRNA, and the protein expression is analyzed with indicated antibodies. (b) BrdU assay to assess the DNA synthesis of A549, A549-shLuc, and A549-shNMU. (c) The percentage of BrdU-positive cells in the A549, A549-shLuc, and A549-shNMU cells (^∗∗∗^*p* < 0.001 vs. A549 or A549-shLuc group). (d) Colony formation assay showing clone formation capacity of A549, A549-shLuc, and A549-shNMU. (e) The number of colonies in each well was counted ^(^^∗∗∗^*p* < 0.001 vs. A549 or A549-shLuc group).

**Figure 8:**
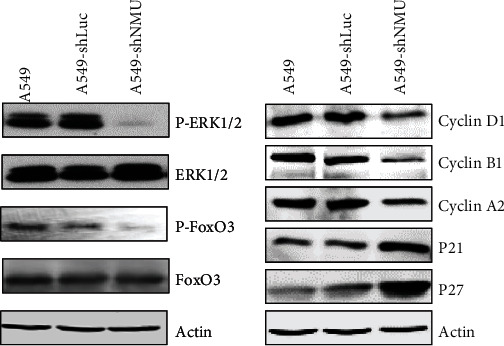
Western blot was for detection of the expression of cAMP signaling and cell cycle-related proteins in A549, A549-shLuc, and A549-shNMU cells.

To explore the impact of NMU on cell proliferation and growth of lung adenocarcinoma, we transfected A549 cells with lentiviruses containing shNMU or shLuc. The protein expression of NMU was repressed after transfection with shNMU (Figure 7(a)). The proliferation of A549 cells was inhibited by treatment with shNMU (Figures 7(b) and 7(c)). Colony formation assays revealed dramatically reduced cell growth after knockdown of NMU (Figures 7(d) an(d) 7(e)). The Erk1/2 and FoxO3 phosphorylation were inhibited in A549 cells treatment with shNMU, suggesting that NMU mainly regulates the cAMP signaling pathway. In addition, cell cycle-associated proteins were determined through western blotting. The data demonstrated that the expression of cyclin A2, cyclin D1, and cyclin B1 decreased in A549 cells transfected with shNMU compared to that transfected with shLuc. Moreover, P21 and P27 expression levels were elevated in A549-shNMU cells than in A549-shLuc cells (Figure 8). The findings demonstrate that NMU plays an indispensable role in the proliferation of LUAD cells and presents a promising treatment target for LUAD.

## 4. Discussion

Studies have shown that bioinformatic methods can be applied to assess gene expression levels and predict potential therapeutic targets [22, 23]. The identification of novel key genes associated with the development and progression of LUAD is crucial for its diagnosis and treatment. Compelling evidence indicates that NMU is upregulated in various cancer types and is related to increased growth and invasion of cancer cells [24–26]. In the present study, we revealed a remarkably higher expression of NMU in LUAD patients than in nonmalignant patients and was associated with LUAD stages I to III. Interestingly, TP53 mutation and cigarette smoking were associated with the high expression of NMU in LUAD patients. The elevated NMU expression was related to the remarkable dismal OS, FPS, and PPS in LUAD patients, suggesting that NMU potentially plays an indispensable role in LUAD prognosis. In addition, through the cBioPortal analysis, we found 3 specific mutations G45V, R143T, and F152L in LUAD patients; the mutations were associated with poor prognosis in LUAD patients. According to the qRT-PCR results, the mRNA expression of NMU was in most cases, overexpressed (3 times higher expression) in the lung adenocarcinoma cells than in nonmalignant lung cells. Both the western blotting and qRT-PCR analyses demonstrated an elevation of the NMU protein expression in lung adenocarcinoma cells. This elevated level of the NMU expression in LUAD tumors was positively correlated with promoting cell proliferation. Moreover, silencing of the NMU expression by RNA interference decreased colony formation. The cAMP signaling cascade mediated the modulation of lung adenocarcinoma cell proliferation by NMU. These findings implicate NMU as a potential new prospective prognostic biomarker and treatment target for LUAD.

Cigarette smoking is a predisposing factor for most lung cancers. Cigarettes have a mixture of over 7000 chemicals with the potential to damage respiratory epithelium and may increase the frequency of genomic alternations [27]. Castelletti et al. revealed that KRAS mutations were linked to smoking [28]. Elsewhere, Mariyo et al. reported that smoking resulted in the MUC4 positive expression and was related to dismal prognosis in patients with LUAD [29]. Herein, we revealed that the LUAD patients who are smoking cigarettes exhibited a higher expression of NMU than their nonsmoking counterparts. Previous studies reported that TP53 was the most predominant mutation in EGFR/KRAS/ALK-negative lung adenocarcinomas in nonsmokers [30]. In addition, TP53 mutations were associated with poor prognosis in LUAD patients. In the present study, we established that TP53 mutations were positively linked to elevated NMU expression in LUAD patients. These results suggested that cigarette smoking and TP53 mutations were related high expression of NMU in LUAD patients.

Moreover, survival analysis illustrated that the high NMU expression was associated with remarkably dismal OS, FPS, and PPS in LUAD patients. The OS revealed that elevated NMU expression resulted in a remarkably low survival probability (HR = 1.7, 95%CI = 15.4 − 2.95, *p* = 0.0074) in LUAD patients. In a previous study, the level of the NMU protein was found to be elevated in hepatocellular carcinoma (HCC), and the prognosis of individuals with HCC with the elevated NMU expression was remarkably worse in contrast with that of individuals with low NMU expression [14]. We also identified the proteins that interacted with NMU via STRING and found 10 NMU interacted proteins: GAL, NPY, NMUR1, NMUR2, GHSR, NPS, MLN, NTS, NTSR1, and NTSR2. Previously, Qiu et al. found remarkably higher levels of NTS, NTSR1, and NTSR2 in colorectal cancer (CRC) tissue than in the surrounding nonmalignant tissue. In addition, they revealed remarkably shorter disease-free survival in patients with CRC with a higher level of NTS than those with lower levels of NTS [31]. Based on the above analysis, we suggest that the NMU interaction with NTS, NTSR1, and NTSR2 is associated with a dismal prognosis in LUAD patients.

KEGG pathway analysis revealed that NMU regulated the cAMP signaling pathway which mediated lung adenocarcinoma development. The cAMP signaling cascade plays an indispensable role in cell growth, cell proliferation, cell differentiation, apoptosis, and metabolism. Yan et al. demonstrated that dopamine and dopamine receptor D1 promoted cell proliferation along with metastasis in hepatocellular carcinoma through the modulation of cAMP/PI3K/AKT/CREB cascade [32]. Ai-wadei et al. found that nicotine stimulated the growth of non-small-cell lung tumor xenografts via the activation of the cAMP signaling cascade. Interestingly, inhibition of cAMP signaling activation reversed the stimulatory influence of nicotine on cancer growth [33]. In this study, we found the high expression of NMU in lung cancer cells activated the cAMP signaling, whereas silencing of NMU inhibited the cAMP signaling activation. Moreover, the cell proliferation and colony formation were reduced in lung adenocarcinoma cells treated with shNMU. The cell cycle-related protein assay demonstrated that silencing NMU decreased cyclin A2, cyclin B1, and cyclin D1 expression, but increased P21 and P27 expression; this implied that NMU knockdown triggered cell cycle arrest and suppressed cell proliferation.

In conclusion, the present study has demonstrated that the NMU expression in LUAD tissues is higher than in nonmalignant tissue. The high NMU expression is a potential specific prognostic biomarker of worse prognosis in individuals with LUAD. Furthermore, NMU regulates cell proliferation through cell cycle and cAMP signaling cascade. Collectively, we have shown that NMU presents a potential prospective prognostic marker and treatment target for LUAD.

## Data Availability

All data generated or analyzed during this study are included in this article.

## References

[1] Wang R., Yamada T., Kita K. (2020). Transient IGF-1R inhibition combined with osimertinib eradicates AXL-low expressing *EGFR* mutated lung cancer. *Nature Communications*.

[2] Denisenko T. V., Budkevich I. N., Zhivotovsky B. (2018). Cell death-based treatment of lung adenocarcinoma. *Cell Death & Disease*.

[3] Wang J., Zou K., Feng X. (2017). Downregulation of NMI promotes tumor growth and predicts poor prognosis in human lung adenocarcinomas. *Molecular Cancer*.

[4] Han X., Luo R., Wang L. (2020). Potential predictive value of serum targeted metabolites and concurrently mutated genes for EGFR-TKI therapeutic efficacy in lung adenocarcinoma patients with *EGFR* sensitizing mutations. *American Journal of Cancer Research*.

[5] Hayashi T., Odintsov I., Smith R. S. (2020). RET inhibition in novel patient-derived models of RET fusion- positive lung adenocarcinoma reveals a role for MYC upregulation. *Disease Models & Mechanisms*.

[6] Zhou Y., Zheng X., Xu B., Deng H., Chen L., Jiang J. (2020). Histone methyltransferase SETD2 inhibits tumor growth via suppressing CXCL1-mediated activation of cell cycle in lung adenocarcinoma. *Aging (Albany NY)*.

[7] Shaurova T., Zhang L., Goodrich D. W., Hershberger P. A. (2020). Understanding lineage plasticity as a path to targeted therapy failure in *EGFR*-mutant non-small cell lung cancer. *Frontiers in Genetics*.

[8] Sun C. C., Zhou Q., Hu W. (2018). Transcriptional E2F1/2/5/8 as potential targets and transcriptional E2F3/6/7 as new biomarkers for the prognosis of human lung carcinoma. *Aging (Albany NY)*.

[9] Lu K., Sui Y., Fu L. (2021). Identification of TRIM56 as a potential biomarker for lung adenocarcinoma. *Cancer Management and Research*.

[10] Tang Q., Li W., Zheng X. (2020). MELK is an oncogenic kinase essential for metastasis, mitotic progression, and programmed death in lung carcinoma. *Signal Transduction and Targeted Therapy*.

[11] Wang Y., Zhou Z., Chen L., Li Y., Zhou Z., Chu X. (2021). Identification of key genes and biological pathways in lung adenocarcinoma via bioinformatics analysis. *Molecular and Cellular Biochemistry*.

[12] Ye Y., Liang Z., Xue L. (2021). Neuromedin U: potential roles in immunity and inflammation. *Immunology*.

[13] Kaushik A. C., Mehmood A., Wei D. Q., Dai X. (2020). Systems biology integration and screening of reliable prognostic markers to create synergies in the control of lung cancer patients. *Frontiers in Molecular Biosciences*.

[14] Li Q., Han L., Ruan S. (2020). The prognostic value of neuromedin U in patients with hepatocellular carcinoma. *BMC Cancer*.

[15] Martinez V. G., O'Neill S., Salimu J. (2017). Resistance to HER2-targeted anti-cancer drugs is associated with immune evasion in cancer cells and their derived extracellular vesicles. *Oncoimmunology*.

[16] Chandrashekar D. S., Bashel B., Balasubramanya S. A. H. (2017). UALCAN: a portal for facilitating tumor subgroup gene expression and survival analyses. *Neoplasia*.

[17] Tang Z., Li C., Kang B., Gao G., Li C., Zhang Z. (2017). GEPIA: a web server for cancer and normal gene expression profiling and interactive analyses. *Nucleic Acids Research*.

[18] Győrffy B., Surowiak P., Budczies J., Lánczky A. (2013). Online survival analysis software to assess the prognostic value of biomarkers using transcriptomic data in non-small-cell lung cancer. *PLoS One*.

[19] Szklarczyk D., Gable A. L., Lyon D. (2019). STRING v11: protein-protein association networks with increased coverage, supporting functional discovery in genome-wide experimental datasets. *Nucleic Acids Research*.

[20] Zhang B., Kirov B. S., Snoddy J. (2005). WebGestalt: an integrated system for exploring gene sets in various biological contexts. *Nucleic acids research*.

[21] Gao J., Aksoy B. A., Dogrusoz U. (2013). Integrative analysis of complex cancer genomics and clinical profiles using the cBioPortal. *Science signaling*.

[22] Sun C. C., Li S. J., Chen Z. L., Li G., Zhang Q., Li D. J. (2019). Expression and prognosis analyses of runt-related transcription factor family in human leukemia. *Molecular Therapy-Oncolytics*.

[23] Sun C. C., Li S. J., Hu W. (2019). Comprehensive analysis of the expression and prognosis for E2Fs in human breast cancer. *Molecular Therapy*.

[24] Lin T. Y., Huang W. L., Lee W. Y., Luo C. W. (2015). Identifying a neuromedin U receptor 2 splice variant and determining its roles in the regulation of signaling and tumorigenesis in vitro. *PLoS One*.

[25] Takahashi K., Furukawa C., Takano A. (2006). The neuromedin U-growth hormone secretagogue receptor 1b/neurotensin receptor 1 oncogenic signaling pathway as a therapeutic target for lung cancer. *Cancer Research*.

[26] Przygodzka P., Papiewska-Pajak I., Bogusz H. (2016). Neuromedin U is upregulated by snail at early stages of EMT in HT29 colon cancer cells. *Biochimica et Biophysica Acta (BBA)-General Subjects*.

[27] Caponnetto P., Caruso M., Maglia M. (2020). Non-inferiority trial comparing cigarette consumption, adoption rates, acceptability, tolerability, and tobacco harm reduction potential in smokers switching to heated tobacco products or electronic cigarettes: study protocol for a randomized controlled trial. *Contemporary clinical trials communications*.

[28] Castelletti N., Kaiser J. C., Simonetto C., Furukawa K., Küchenhoff H., Stathopoulos G. T. (2019). Risk of lung adenocarcinoma from smoking and radiation arises in distinct molecular pathways. *Carcinogenesis*.

[29] Rokutan-Kurata M., Yoshizawa A., Sumiyoshi S. (2017). Lung adenocarcinoma with MUC4 expression is associated with smoking status, HER2 protein expression, and poor prognosis: clinicopathologic analysis of 338 cases. *Clinical Lung Cancer*.

[30] Ahn J. W., Kim H., Yoon J. K. (2014). Identification of somatic mutations in EGFR/KRAS/ALK-negative lung adenocarcinoma in never-smokers. *Genome Medicine*.

[31] Qiu S., Nikolaou S., Zhu J. (2020). Characterisation of the Expression of Neurotensin and Its Receptors in Human Colorectal Cancer and Its Clinical Implications. *Biomolecules*.

[32] Yan Y., Pan J., Chen Y. (2020). Increased dopamine and its receptor dopamine receptor D1 promote tumor growth in human hepatocellular carcinoma. *Cancer Communications*.

[33] AN Al-Wadei H., Al-Wadei M. H., Ullah M. F., Schuller H. M. (2012). Gamma-amino butyric acid inhibits the nicotine-imposed stimulatory challenge in xenograft models of non-small cell lung carcinoma. *Current Cancer Drug Targets*.

